# Specificity of transcranial sonography in parkinson spectrum disorders in comparison to degenerative cognitive syndromes

**DOI:** 10.1186/1471-2377-12-12

**Published:** 2012-03-08

**Authors:** Kristina Laučkaitė, Daiva Rastenytė, Danguolė Šurkienė, Antanas Vaitkus, Andrius Sakalauskas, Arūnas Lukoševičius, Rymantė Gleiznienė

**Affiliations:** 1 Department of Neurology, Lithuanian University of Health Sciences, Academy of Medicine, Mickevičiaus street 9, Kaunas, Lithuania; 2 Biomedical Engineering Institute, Kaunas University of Technology, Studentų street 65, Kaunas, Lithuania; 3 Department of Radiology, Lithuanian University of Health Sciences, Academy of Medicine, Mickevičiaus street 9, Kaunas, Lithuania

**Keywords:** Transcranial sonography, Substantia nigra, Parkinson's disease, Cognitive disorders, Specificity, Sensitivity

## Abstract

**Background:**

Hyperechogenicity of the substantia nigra (SN+), detected by transcranial sonography (TCS), was reported as a characteristic finding in Parkinson's disease (PD), with high diagnostic accuracy values, when compared mainly to healthy controls or essential tremor (ET) group. However, some data is accumulating that the SN + could be detected in other neurodegenerative and even in non-neurodegenerative disorders too. Our aim was to estimate the diagnostic accuracy of TCS, mainly focusing on the specificity point, when applied to a range of the parkinsonian disorders, and comparing to the degenerative cognitive syndromes.

**Methods:**

A prospective study was carried out at the Hospital of Lithuanian University of Health Sciences from January until September 2011. Initially, a TCS and clinical examination were performed on 258 patients and 76 controls. The General Electric Voluson 730 Expert ultrasound system was used. There were 12.8% of cases excluded with insufficient temporal bones, and 4.3% excluded with an unclear diagnosis. The studied sample consisted of the groups: PD (n = 71, 33.2%), ET (n = 58, 27.1%), PD and ET (n = 10, 4.7%), atypical parkinsonian syndromes (APS) (n = 3, 1.4%), hereditary neurodegenerative parkinsonism (HDP) (n = 3, 1.4%), secondary parkinsonism (SP) (n = 23, 10.8%), mild cognitive impairment (MCI) (n = 33, 15.4%), dementia (n = 13, 6.1%), and control (n = 71).

**Results:**

There were 80.3% of PD patients at stages 1 & 2 according to Hoehn and Yahr. At the cut-off value of 0.20 cm^2 ^of the SN+, the sensitivity for PD was 94.3% and the specificity - 63.3% (ROC analysis, AUC 0.891), in comparison to the rest of the cohort. At the cut-off value of 0.26 cm^2^, the sensitivity was 90% and the specificity 82.4%.

The estimations for the lowest specificity for PD, in comparison to the latter subgroups (at the cut-off values of 0.20 cm^2 ^and 0.26 cm^2^, respectively) were: 0% and 33.3% to APS, 33.3% and 66.7% to HDP, 34.8% and 69.6% to SP, 55.2% and 82.8% to ET, 75% and 91.7% to dementia.

**Conclusions:**

The high sensitivity of the test could be employed as a valuable screening tool. But TCS is more useful as a supplementary diagnostic method, due to the specificity values not being comprehensive.

## Background

The burden of progressive neurodegenerative diseases in the elderly population is continuously growing, emphasizing the need of improving diagnostics and treatment [1]. Parkinson's disease (PD) is the second most prevalent neurodegenerative disease after Alzheimer's disease (AD) [1]. However, the diagnosis of PD or AD is based solely on the clinical criteria, as there is no accurate biological or imaging marker to date for either of them [1-3].

Single photon emission computed tomography (SPECT) is the most widely used diagnostic tool in parkinsonian disorders. In PD, there is a diminished uptake of the ligand, which usually correlates with the clinically affected side, and with progression of PD [2]. But SPECT does not differentiate between PD and other causes of parkinsonism [2]. If the presynaptic ligands are used, it raises the sensitivity, but not the specificity for PD and syndromes [3,4]. Besides, SPECT is not widely available and limited in terms of safety [2].

Both magnetic resonance imaging (MRI) and computed tomography (CT) are used to evaluate the structural changes of the brain. These imaging methods are not usually needed for the diagnosis, but find their place and value if parkinsonism is purely unilateral or otherwise atypical, which exclude secondary causes [3]. Both MRI and CT have their own limitations related to availability, costs, contraindications and safety (CT) [2,3].

Among the new techniques, transcranial B-mode duplex sonography (TCS) has been drawing a lot of attention as an easily accessible and inexpensive imaging method [5]. TCS is sharing some features of the functional imaging methods, which is thought to detect very early dysfunction of the nigrostriatal pathways [6]. This probably demonstrates an increased vulnerability of the extrapyramidal system, or even an increased risk of PD development [6,7].

**Figure 1:**
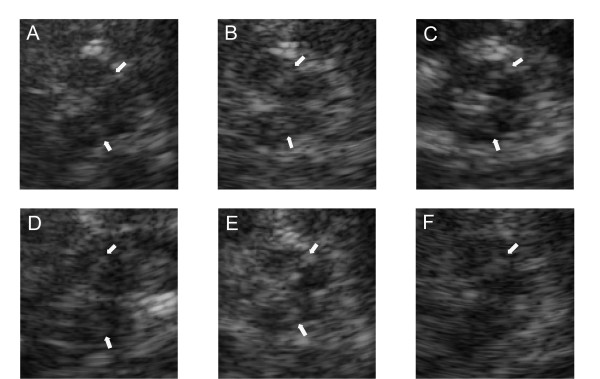
**Hyperechogenicity of the SN detectable in various diseases, other than PD (.tif)**. TCS with the SN+ (hyperintensive signal marked with white arrows) in a patient with essential tremor, A- in a patient with Stiff person syndrome, C- in a healthy control, D- in a case of progressive supranuclear palsy, E- in a severely depressed subject, and F- in secondary (vascular) parkinsonism. All the pictures are made in our laboratory with GE Voluson 730 Expert ultrasound system, with the permissions gained from all the patients to reproduce the images confidentially.

Hyperechogenicity of the SN (SN+), both in echo intensity and size of area, has been repeatedly reported as a characteristic TCS finding in PD patients. But it can be also detected in some other neurodegenerative and even in non-neurodegenerative disorders [8,9], or related to aging [7,10,11]. Therefore, the issue regarding sensitivity and specificity of TCS is still disputable (see Figure 1 for the illustration of the observed SN+ in disorders other than PD in our study). While many studies have estimated specificity of TCS comparing PD patients to healthy controls or essential tremor (ET) group [5,12-22], to date, very few case control studies involved patients with atypical parkinsonian syndromes (APS), neurodegenerative hereditary (HDP) or secondary parkinsonism (SP) [4,23-27].

The aim of the present study was to estimate the diagnostic accuracy of TCS, mainly focusing on the specificity point. TCS findings in PD patients were compared to the controls and to the findings in patients with variety of parkinsonian syndromes, ET, mild cognitive impairment (MCI) and dementia groups.

## Methods

### Enrolment of the patients and controls

The patients for TCS investigation were referred from both Out-patient (n = 156, 72.9%) and In-patient (n = 58, 27.1%) units of Neurological Department of the Hospital of Lithuanian University of Health Sciences (Kaunas, Lithuania). The study was conducted from January 2011 till September 2011. Initially, a total of 258 adult patients with extrapyramidal symptoms and signs were invited to take part in the study. From a total sample, we had to exclude 33 (12.8%) of the subjects because of bilateral temporal acoustic bone insufficiency. For 11 (4.2%) of the patients, the diagnosis was still not clear after a six month follow-up period. Therefore 214 patients were enrolled for further analysis.

The control group consisted of 71 matched-to-PD group individuals, who were without signs of movement or cognitive disorders. They were referred for extracranial artery duplex ultrasound scanning.

A clinical diagnosis after a six month follow-up was chosen as a gold diagnostic standard for PD [3]. When the diagnosis remained unclear clinically, or TCS results were doubtful and not consistent with the clinical findings, SPECT with presynaptic ^123^I labelled Ioflupane ligand was employed. For the patients with pyramidal signs or other atypical symptoms, brain CT or MRI scans were performed by experienced neuroradiologist (RG).

The 214 patients group consisted of 71 (33.2%) PD patients (meeting the criteria of the United Kingdom Parkinson's Disease Society Brain Bank [28], 58 (27.1%) ET patients, 10 (4.7%) patients with signs and symptoms of both PD and ET (a combined form), and 23 (10.8%) patients to whom secondary parkinsonism (SP) was diagnosed. In addition, there were 3 (1.4%) patients with atypical parkinsonian syndrome (APS), 3 (1.4%) patients with hereditary neurodegenerative parkinsonism (HDP), 33 (15.4%) patients with mild cognitive impairment (MCI), and 13 (6.1%) with dementia.

The entire spectrum of parkinsonian syndromes, which were tested, consisted of APS (2 patients with progressive supranuclear palsy and 1 with multisystem atrophy), HDP (1 patient with early-onset Huntington's disease (HD), 2 patients with Wilson's disease (WD)), SP (4 patients with hydrocephalus, 2 patients with extrapyramidal signs of vascular origin, 2 patients with tumor, 2 post-traumatic patients, 1 post-infective patient, 1 psychogenic, 1 patient with toxins-induced parkinsonism, 1 patient with drugs induced parkinsonism, 1 patient with amyotrophic lateral sclerosis, and 8 patients of undefined etiology). We included the patients both with clinically suspected (de novo) parkinsonian spectrum disorders (n = 104, 61.9%), and with clinically well defined diagnoses, the latter having already had prescribed treatment (n = 64, 38.1%).

The dementia group consisted of 6 (46.2%) patients with Alzheimer's disease, 6 (46.2%) patients with vascular dementia, and 1 (7.6%) patient with a not yet determined dementia type.

Before the study was initiated, permission from the Regional Ethical Committee of Kaunas (Lithuania) was obtained. All participants gave written informed consent according to the Declaration of Helsinki.

### Clinical and instrumental approach

The study had a prospective manner. A TCS, clinical examination (mainly extrapyramidal tone of the muscles, tremor and/or bradykinesia, its location), a structural interview (family history, head traumas, symptom duration, type of a job, intoxications, most prominent motor and non-motor symptoms at the time of examination), also subjective (Hospital Anxiety and Depression Scale (HAD) [29] and objective scales (the Unified Parkinson's Disease Rating Scale (UPDRS) (DS) [30] were employed. The Mini Mental State Examination (MMSE) [31] and the Alzheimer's Disease Assessment Scale- Cognitive (ADAS Cog) [32] were used for the cognitive assessment.

### Methods of TCS

A PA2-5, wide band, phased array sector transducer (*f_0 _*= 1.4 MHz, band with 1.3 - 4 MHz, footprint 20 × 14 mm (128 elements)), on the commercially available ultrasound system, Voluson 730 Expert (General Electric Healthcare, Austria), was used for all examinations. At first, a calibration of the ultrasound machine with a phantom was performed, cooperating with the scientists from the University of Technology of Kaunas (AS, AL) [33]. The parameters of the ultrasound system were chosen as recommended by Walter U et al. [34]. The transducer was placed over the pre-auricular temporal acoustic window on both sides of the patients and controls. By laying the patients and healthy subjects in a supine position in a darkened room, and by focusing at a depth of 16.8 cm/55 Hz, at a power of 100 W, we achieved optimal image quality of the structures at the midbrain level and at other planes. The overall gain and degree of compression (TGC curve) were manually adapted if necessary, with a lateral gain control of the machine. A mechanical index of 0.7 and a thermal index of 0.3 were ensured according to recommendations for the safe use of diagnostic ultrasound equipment. TCS on all of the participants was performed by one of the authors (KL), who was blind to the initial diagnosis of each patient and control. The sequences of TCS images of all subjects were obtained in AVI format. Each sequence consisted of approximately 200 frames (size of single frame 600 × 800 pixels, single pixel size 0.0421 cm). After freezing the best image for the measurements (the largest plot of the SN), it was zoomed three-fold. Within the mesencephalon, the hyperechogenic signal of the ipsilateral SN to transducer was measured, along with other structures on the contralateral side. Video footage was acquired using 2D cine scanning mode, and was stored for reviews after TCS examination.

### Parameters of TCS analysis

The SN+ was recorded in echo intensity (using qualitative 5° visual grading) and in size of area (planimetry, outlined manually in cm^2^). Grade I of the SN echogenicity was defined as the same echogenicity as the brainstem, grade II - when clearly detectable from the brainstem, grade III- lower signal intensity when comparing to perimesencephalic cisterns, grade IV - the same to perimesencephalic cisterns, grade V - higher than perimesencephalic cisterns [35].

For the quantitative evaluation, we calculated the mean value of the SN echogenicity, from the measurements of the healthy controls, as 0.14 ± 0.06 cm^2 ^(mean ± 1 SD). We followed the recommendations of Berg D et al., to use the mean + 1 or 2 SD as an upper norm value [36]. We treated the SN+ if at least one side was enlarged, and the enlargement was by > 0.20 cm^2^.

Together with the SN, other TCS parameters were recorded. That included the diameters of the third and lateral ventricles, and the echogenicity of the raphe, red, lentiform, caudate nuclei and of the central grey matter.

### Statistical analysis

A database was created using Microsoft Office Excel 2003. For the statistical analysis, data was exported to the statistical package IBM SPSS version 19 (IBM, USA). For the two group comparison of unpaired, non-parametric data, Mann-Whitney *U *test and Fisher's exact tests were used. For three or more groups, the Kruskal-Wallis H test was used. For parametric data, analysis of variance (ANOVA) was used, for the comparison between three or more groups. Where appropriate, the corrections for multiple comparisons were performed. Post hoc tests of Tukey and LSD were performed where necessary, to obtain the exact differences between the groups by multiple comparisons. For categorical data, *χ*^2 ^criterion was used. The cut-off value was established by Receiver Operating Characteristics (ROC) analysis. Correlation analysis of non-parametrical data was performed by Spearman's rank correlation. The P value of less than 0.05 was used as the criterion for statistical significance.

## Results

### Sample characteristics

Due to permeable acoustic temporal bone windows being on both sides, bilateral investigation was performed on 141 (65.9%) of the 214 TCS examination patients. Unilateral investigation was carried out on the remaining 73 (34.1%) patients. The majority of the excluded patients (n = 33, 12.8%) with insufficient temporal acoustic bones were of a female gender (91.9%), with only 8.1% being male. According to the age, they were of 70.7 ± 7.8 years.

A summary of demographic and clinical characteristics of the patients (n = 214) and controls (n = 71), is given in additional file [see Additional file 1]. Statistical findings from multiple comparisons revealed that PD patients were significantly younger compared to the dementia group (63.8 ± 10.1 years vs. 71.0 ± 11.8 years, respectively), were of predominant male gender (57.8%), and 90.1% with detectable raised extrapyramidal tone. There was positive family history for 14.1% of PD patients, compared to 34.5% from the ET group. A quite significant part (16.9%) mentioned contact with toxic agents at work in the PD group.

The different clinical subtypes of PD were as follows: 20 (28.1%) patients were tremor-predominant, 19 (26.8%) were bradykinesia/akinesia predominant and 32 (45.1%) had a mixed type of the disease. According to a relative level of disability (disease stage by Hoehn and Yahr scale), the patients were classified as having stage 1-22 (31.0%), stage 2-35 (49.3%), stage 3-13 (18.3%), and stage 4-1 (1.4%) in the PD group. The median symptom duration was 3 years. The UPDRS part III scores for PD patients were (mean ± SD) 12.63 ± 1.73 points (a range from 4 to 23 points).

The lowest cognitive scores were counted for the SP (MMSE 18.0 ± 2.8 points) and dementia (MMSE 20.6 ± 2.4 points) groups. The Adas Cog scores differed statistically significantly between MCI and dementia groups (17.9 ± 4.7 vs. 29.2 ± 8.1 points).

### TCS findings

The results of TCS examination are presented in additional file [see Additional file 2]. The SN+ was detected in 66 (92.96%), and it was negative to 5 (7.04%) patients of the PD group. The SN+ was present among the majority of PD patients bilaterally (n = 51, 77.3%). We detected a high percentage of the SN+ at a threshold of 0.20 cm^2 ^in other parkinsonian spectrum disorders (70% in PD plus ET, 66.7% in APS, 52.1% in SP, 33.3% in HDP groups). In the ET group it was 31%. When compared to the control group, a higher frequency of the SN+ was detected in the dementia group (15.6% vs. 11.2%, respectively).

The SN greatest plot between the groups quantitatively was then compared. Tukey post hoc analysis revealed, that it did not differ the plot of the PD group from PD plus ET group (*p *= 0.995), APS (*p *= 0.378) and HDP (*p *= 0.348). But it demarcated clearly from the rest of the groups (ET, SP, MCI, dementia and controls).

The qualitative measures of the SN in the PD group were successively distributed as grade I- 0 (0%), grade II- 5 (7.4%), grade III- 32 (47.1%), grade IV- 30 (44.1%), grade V- 1 (1.5%). A positive correlation was found between the intensity of the SN signal (grade), and the plot (cm^2^) in all the patient groups (Spearman's rho = 0.676, *p *< 0.001).

In the PD group, the mean plot of the SN+ differed in each of the clinical subtypes of the disease. This was the largest in the hypokinetic/akinetic 0.47 ± 0.16 cm^2 ^(ANOVA, F = 4.13, *p *= 0.01) variant. A statistically significant correlation was detected between the hyperechogenic plot of the SN on the right side (*p *= 0.02), but not on the left side (*p *= 0.06), with contralateral clinical symptoms at onset of PD.

A diameter of the third ventricle differed statistically significantly between the PD and SP groups (*p *= 0.04), and also between the PD and dementia groups (*p *= 0.02). The diameter of the right lateral ventricle also differed significantly between the PD and MCI (*p *= 0.04), and when compared to the dementia group (*p *= 0.03). Taking into account the results from all the groups, we found that the diameter of the third and of the lateral ventricles correlated positively with age (Spearman's rho = 0.494, *p *< 0.001, and Spearman's rho = 0.233, *p *= 0.002, respectively).

### Specificity and sensitivity of TCS

The cut-off value for the plot of the SN was established using ROC curve with the sensitivity of 94.3% for PD, which corresponded to an area of 0.20 cm^2 ^(AUC 0.891), and at this cut-off value the specificity was 63.3%. When the cut-off value reached 0.26 cm^2^, the sensitivity was 90% and the specificity 82.4%, when comparing the PD group to the rest of our cohort.

When separately comparing the PD group to the ET group, ROC analysis revealed that at the cut-off value of 0.20 cm^2^, the diagnostic specificity was 55.2%, and at the cut-off value of 0.26 cm^2 ^it was 82.8% (AUC 0.903) (see Table 1 - Diagnostic accuracy of TCS for PD).

**Table 1:** Diagnostic accuracy of TCS for PD

Accuracy	Cut-off value (cm^2^)	Parkinson spectrum disorders					Cognitive disorders		Control n = 71
		
		PD n = 71	ET n = 58	APS n = 3	HDP n = 3	SP n = 23	MCI n = 33	D n = 13	
Se (%)	0.20	94.3	N/A	N/A	N/A	N/A	N/A	N/A	N/A
	
	0.26	90	N/A	N/A	N/A	N/A	N/A	N/A	N/A

Sp (%)	0.20	N/A	55.2	0	33.3	34.8	89.7	75	83.3
	
	0.26	N/A	82.8	33.3	66.7	69.6	93.1	91.7	95.8

Abbreviations: *N/A *not applicable, *TCS *transcranial sonography, *Se *sensitivity, *Sp *specificity, *PD *Parkinson's disease, *ET *essential tremor, *APS *atypical parkinsonian syndromes, *HDP *hereditary degenerative parkinsonism, *SP *secondary parkinsonism, *MCI *mild cognitive impairment, *D *dementia

Then the PD group was compared to other parkinson spectrum disorders individually. The lowest diagnostic specificity for PD of 0% to 33.3% was estimated at both the cut-off values of 0.20 cm^2 ^and 0.26 cm^2 ^(AUC 0.903) respectively, when compared to APS. However, the third ventricle diameter above 10 mm raised the diagnostic specificity (vs. APS) up to 66.6%. The same was reached when comparing the PD group to the SP group. The diameter of the third ventricle greater than 10 mm raised the specificity up to 78.3%.

When compared the PD group to the cognitive syndromes, the specificity was estimated much lower (75%), when compared to the dementia group at the cut-off value of 0.20 cm^2 ^(AUC 0.905).

## Discussion

Our study addressed the important issue of the sensitivity and specificity of TCS to determine clinical diagnosis of PD. It is a matter of concern about false positives (the subjects with the SN+ detected by TCS, but without PD) in screening tests, as we don't want to tell someone that they have a serious disease when they do not really have it. The specificity of the TCS refers to the ability of the test to correctly identify those patients without PD. In this pilot study, by ROC analysis, we calculated the sensitivity of TCS for PD 94.3%, and the specificity 63.3% at the cut-off value of 0.20 cm^2^, when compared to the rest of the cohort. While at the cut-off value of 0.26 cm^2^, the sensitivity was calculated 90%, and the specificity 82.4%.

The cut-off values, developed in our laboratory for the SN+ threshold of 0.20 cm^2 ^[4,12-14,17,20,21,24,25] and of 0.26 cm^2 ^[37], were consistent with the findings of other authors. The new feature of our study was the different ultrasound system, which was not employed and reported by previous studies, confirming that data acquired by this device is comparable to other ultrasound systems.

The specificity was markedly lower, when in comparison to the results, published by some other authors [14,25-27]. This finding raised issues on the diagnostic specificity of TCS in the differentiation of Parkinson spectrum disorders. SPECT has the same limitations, when performed with the presynaptic ligands [2-4]. It was especially evident when comparing the PD to APS (33.3%), to HDP (66.7%) and to SP (69.6%) groups, even at the cut-off value of 0.26 cm^2^. Although in a less prevalent manner, the SN+ could be detected in some other neurodegenerative diseases, as well as in some subgroups of the patients with non-degenerative diseases and in disorders associated with an increased risk of PD [38]. If it is known that the SN+ in these disorders is also present already at the preclinical stages, this might contribute to a lower diagnostic specificity [9].

It is important to emphasize that the values of diagnostic accuracy were counted solely on the SN measurements, and the integration of the other TCS features (the diameters of the ventricles, echogenicity of other basal ganglia) proved helpful. We noticed the common signs detected by TCS in PD patients were: 1) more frequent bilateral involvement of the SN (77.3%), 2) evidently a much larger area of the SN+ (mean 0.42 cm^2 ^in the PD group, while in APS, HDP, SP approximately 0.25 cm^2^), 3) with a mean third ventricle diameter of 0.58 cm (similar to the ET and MCI groups), 4) an absent or interrupted nuclei raphe in the majority of cases, and 5) the moderately increased central grey matter. We introduced the latter parameter (a potentially useful ultrasound marker), as this area is usually evaluated during MRI studies in parkinsonian syndromes and resembles possible periaqueductal gliosis. The central grey matter was the most evidently hyperechogenic in dementia, APS and HDP groups.

A comparison to the degenerative cognitive syndromes also added some new information. The lower diagnostic specificity for PD when compared to the dementia group (75% at the cut-off value 0.20 cm^2 ^of the SN) should be evaluated critically, as one patient of the group exhibited clearly detectable extrapyramidal tone of the muscles, two of the patients- subtle slow movements, proving possible involvement of extrapyramidal nervous system in the pathologically aging brain. The main TCS parameters, which enabled to raise diagnostic specificity, were the significantly wider diameters of the third and lateral ventricles in the dementia group.

It is necessary to emphasize that in our study the majority of PD patients were at the early stage of the disease (80.3%), and of the de novo diagnosis (61.9%). However, some of the patients fell into different categories where it was not possible to clearly distinguish PD and ET after 6 months of the follow-up period. Those patients, along with the patients, who had totally unclear diagnosis, were excluded from any further analysis. This could have a positive influence on the values of the diagnostic accuracy. Another limitation of the study was finding a small number of cases in APS and HDP groups. We have chosen the control group from the subjects, who were referred to the vessel ultrasound, who were suspected to have vascular disorders. The main limitation of the TCS method itself was that there was a high number of bilateral temporal acoustic bone insufficiency found (12.8%), mainly among older female patients.

We have found the highest plots of the SN+ in the hypokinetic/akinetic PD type. This feature is in parallel with the results, where the SN+ correlated with the bilateral bradykinesia and rigidity [35]. The SN+ on the right side was larger to the contralateral clinical symptoms on disease presentation (*p *= 0.021), but was not replicated on the left side to the contralateral clinical symptoms (*p *= 0.055). This was consistent with some other studies, where signal extension was usually larger contralaterally to the more clinically affected side [36,37].

## Conclusions

The lowest specificity of the TCS for PD was detected by making comparisons to the other parkinsonian syndromes, ET and dementia. The sensitivity was high (94.3% and 90% at the cut-off values of 0.20 cm^2 ^and 0.26 cm^2^, respectively), based solely on the measures of the SN area, and taking into account the early stages of PD and the heterogeneous sample. The high sensitivity of the test could be employed as a valuable screening tool for PD, but is better to be used as a supplementary diagnostic method, due to the specificity values not being comprehensive. However, the diagnostic specificity could be raised by combining other TCS parameters, the results of clinimetric scales, clinical information and other imaging tests.

## Competing interests

The authors declare that they have no competing interests.

## Authors' contributions

KL performed TCS, acquired the data, contributed to analysis and interpretation of the data, and wrote the first draft of the manuscript. DŠ examined the patients neurologically, also applied objective clinimetric scales and collected the data. RG performed brain CT and MRI scans for the patients to rule out secondary causes, and contributed to the interpretation of the data in comparison to TCS. AS and Professor AL were responsible for the technical improvement of the ultrasound system, as well as the acquisition and statistical analysis of the data. Professor DR was responsible for the main idea and design of the study, and together with associated professor AV revised the manuscript critically, and have given the final approval of this version to be published. All authors have read and approved the final manuscript.

## Pre-publication history

The pre-publication history for this paper can be accessed here:

http://www.biomedcentral.com/1471-2377/12/12/prepub

## Supplementary Material

Additional file 1 **Demographic and clinical characteristics of the patients and controls (Table 2.doc)**. There are given characteristics of all the studied subgroups of the patients, and control subjects in detail. After the six months of follow-up, we grouped the sample of patients into parkinson spectrum disorders and patients with cognitive deficits. The statistical differences were counted. Click here for file

Additional file 2 **Results of TCS (Table 3.doc)**. Presented in this table are all the results, gained after performing TCS with the statistically counted differences. The measurements were presented according to the groups of PD patients and controls. Click here for file
