# Supplementary material for: Specificity of transcranial sonography in parkinson spectrum disorders in comparison to degenerative cognitive syndromes
Source: BMC Neurol. 2012 Mar 8;12:12. doi: 10.1186/1471-2377-12-12 (PMC3317847; doi:10.1186/1471-2377-12-12)
Supplement: Additional file 1 — Demographic and clinical characteristics of the patients and controls (Table 2.doc). There are given characteristics of all the studied subgroups of the patients, and control subjects in detail. After the six months of follow-up, we grouped the sample of patients into parkinson spectrum disorders and patients with cognitive deficits. The statistical differences were counted. [file 1471-2377-12-12-S1.DOC]

| Demographic and clinical characteristics | Parkinson spectrum disorders | | | | | | Cognitive disorders | | Control group  (n=71) |
| --- | --- | --- | --- | --- | --- | --- | --- | --- | --- |
|  | PD  (n=71) | ET  (n=58) | PD and ET  (n=10) | APS  (n=3) | HDP  (n=3) | SP  (n=23) | MCI  (n=33) | Dementia  (n=13) |  |
| Age, y, mean± SD^A^ | 63.8±10.1 | 63.5± 12.6 | 70.2± 8.4 | 67.3± 9.7 | 55.7± 4.2 | 66.4± 12.5 | 68.1± 10.5 | 71.0± 11.8 | 61.5± 13.3 |
| Gender, male/female, n | 41/30 | 23/35 | 5/5 | 2/1 | 2/1 | 13/10 | 6/27 | 3/10 | 41/30 |
| Family history +, n (%) | 10 (14.1) | 20 (34.5) | 3 (30) | 0 (0) | 1 (33.3) | 1 (4.4) | 7 (21.2) | 0 (0) | 2 (0.03) |
| Head traumas +, n (%) | 8 (11.3) | 14 (24.1) | 1 (10) | 0 (0) | 1 (33.3) | 4 (17.4) | 7 (21.1) | 3 (23.1) | 5 (7) |
| Toxic exposure +, n (%) | 12 (16.9) | 8 (13.8) | 3 (30) | 1 (33.3) | 0 (0) | 3 (13) | 4 (12.1) | 1 (7.7) | 3 (4.2) |
| Symptoms duration, y, median (IQR)^B^ | 3 (2-6) | 5 (2-11) | 8 (5-16) | 5 (0) | 10 (0) | 1 (0.3-3) | 2 (1-4) | 2 (2-4) | 0.1 (0-2) |
| H-Y stage, median (IQR)^C^ | 2 (1-2) | N/A | 2 (0-3) | N/A | N/A | N/A | N/A | N/A | N/A |
| MMSE points, mean± SD^D^ | 24.1± 3.7 | 26.8± 1.5 | N/A | 26.0 | N/A | 18.0± 2.8 | 26.0± 2.2 | 20.6± 2.4 | 27.6± 3.6 |
| ADAS Cog scores, mean± SD^E^ | N/A | N/A | N/A | N/A | N/A | N/A | 17.9± 4.7 | 29.2± 8.1 | N/A |
| HAD scale, A/D mean scores | 6.9/6.0 | 6.0/3.8 | N/A | N/A | N/A | 2.5/5.5 | 7.8/8 | N/A | 9.0/5.3 |
| Extrapyramidal tone +, n (%) | 64 (90.1) | 2 (3.5) | 6 (60) | 1 (33.3) | 0 (0) | 11 (47.8) | 0 (0) | 1 (7.7) | 0 (0) |

1. ANOVA, F= 2.13, p=0.03; multiple comparisons by post hoc test of LSD revealed statistically significant differences between PD and dementia groups (p=0.04), ET and dementia (p=0.03), PD+ET and controls (p=0.02), MCI and controls (p=0.01), dementia and controls (p=0.01), HDP and dementia (p=0.04).
2. Kruskal-Wallis H test, χ2=83.2, p<0.001.
3. Mann-Whitney U test, Z=-0.18, p=0.86.
4. ANOVA, F=13.6, p<0.001.
5. ANOVA, F=10.5, p<0.001.

Abbreviations: PD- Parkinson’s disease, ET- essential tremor, APS- atypical parkinsonian syndromes, HDP- hereditary degenerative parkinsonism, SP- secondary parkinsonism, MCI- mild cognitive impairment, y- years, SD- standard deviation, IQR- interquartile range, presented as Q1- Q3, H-Y- Hoehn- Yahr scale, MMSE- Mini Mental State Exam, ADAS Cog- Alzheimer’s disease Assessment Scale, Cognitive Subscale, A/D- anxiety/depression components, N/A- not applicable or not applied.
