# Supplementary material for: Specificity of transcranial sonography in parkinson spectrum disorders in comparison to degenerative cognitive syndromes
Source: BMC Neurol. 2012 Mar 8;12:12. doi: 10.1186/1471-2377-12-12 (PMC3317847; doi:10.1186/1471-2377-12-12)
Supplement: Additional file 2 — Results of TCS (Table 3.doc). Presented in this table are all the results, gained after performing TCS with the statistically counted differences. The measurements were presented according to the groups of PD patients and controls. [file 1471-2377-12-12-S2.DOC]

| Measurements by TCS | Parkinson spectrum disorders | | | | | | Cognitive disorders | | Control group (n=71) |
| --- | --- | --- | --- | --- | --- | --- | --- | --- | --- |
|  | PD (n=71) | ET  (n=58) | PD and ET (n=10) | APS  (n=3) | HDP  (n=3) | SP  (n=23) | MCI (n=33) | Dementia (n=13) |  |
| SN_R_, cm^2^, mean± SD^A^ | 0.34± 0.14 | 0.17± 0.09 | 0.24± 0.16 | 0.23± 0.03 | 0.2± 0.17 | 0.21± 0.11 | 0.14± 0.08 | 0.13± 0.06 | 0.14± 0.06 |
| SN_L_, cm^2^, mean± SD^B^ | 0.39± 0.14 | 0.19± 0.12 | 0.36± 0.18 | 0.22± 0.08 | 0.21± 0.07 | 0.24± 0.15 | 0.13± 0.05 | 0.19± 0.18 | 0.14± 0.06 |
| SN_Max._, cm^2^, mean± SD^C^ | 0.42± 0.14 | 0.21± 0.11 | 0.38± 0.19 | 0.25± 0.05 | 0.25± 0.13 | 0.25± 0.12 | 0.15± 0.07 | 0.18± 0.16 | 0.16± 0.06 |
| SN+ ≥0.20 cm^2^, n (%)^D^ | 3 (4.2) | 8 (13.8) | 0 (0) | 0 (0) | 0 (0) | 5 (21.7) | 0 (0) | 1 (7.8) | 5 (7) |
| SN+ ≥0.26 cm^2^, n (%)^D^ | 63 (88.7) | 10 (17.2) | 7 (70) | 2 (66.7) | 1 (33.3) | 7 (30.4) | 2 (6.1) | 1 (7.8) | 3 (4.2) |
| III ventricle, cm, mean± SD^E^ | 0.58± 0.28 | 0.6± 0.27 | 0.68± 0.14 | 0.82± 0.43 | 0.9± 0.17 | 0.78± 0.31 | 0.57± 0.32 | 0.73± 0.35 | 0.49± 0.26 |
| LV_R_, cm, mean± SD^F^ | 1.79± 0.29 | 1.88± 0.23 | 1.82± 0.29 | 1.77± 0.8 | 1.63± 0.1 | 2.06± 0.48 | 1.81± 0.25 | 2.15± 0.27 | 1.59± 0.6 |
| LV_L_ cm, mean± SD^G^ | 1.47± 0.68 | 1.35± 0.87 | 1.08± 0.94 | 1.10± 0.95 | 1.15± 1.0 | 1.39± 1.01 | 1.11± 0.86 | 1.08± 1.06 | 1.13± 0.9 |
| N. ruber +, n (%) | 2 (2.8) | 5 (8.6) | 1 (10) | 0 (0) | 0 (0) | 1 (4.3) | 3 (9.1) | 0 (0) | 11 (15.5) |
| N. raphe absent/partial, n (%)^H^ | 60 (84.5) | 21 (36.2) | 7 (70) | 2 (66.7) | 2 (66.7) | 14 (60.9) | 18 (54.5) | 6 (46.2) | 15 (21.1) |
| N. lentiformis +, n (%)^I^ | 4 (5.6) | 6 (24.0) | 3 (12.0) | 0 (0) | 1 (33.3) | 5 (21.7) | 2 (6.1) | 1 (7.7) | 1 (1.4) |
| S. grisea centralis +, n (%)^J^ | 19 (26.8) | 14 (24.1) | 3 (30) | 3 (100) | 2 (66.7) | 5 (21.7) | 5 (15.2) | 6 (46.2) | 12 (16.9) |

^A^- ANOVA, F=18.9, p<0.001, multiple comparisons by post hoc analysis of Tukey test revealed significant differences between PD and ET, SP, MCI, dementia and controls (p<0.001).

^B^- ANOVA, F=22.6, p<0.001, multiple comparisons by post hoc analysis of Tukey test revealed significant differences between PD and ET, SP, MCI, dementia and controls (p<0.001).

^C^- ANOVA, F=27.5, p<0.001, multiple comparisons by post hoc test of Tukey revealed significant differences between PD and ET, SP, MCI, dementia and controls (p<0.001).

^D^- When the substantia nigra (SN) hyperechogenicity plot ≥0.20 cm^2^ (moderate) and ≥0.26 cm^2^ (marked).

^E^-ANOVA, F=2.54, p=0.011, post hoc analysis of LSD revealed significant differences between PD and SP (p=0.04), dementia (p=0.02).

^F^- ANOVA, F=3.24, p=0.002, post hoc analysis of LSD revealed significant differences between PD and MCI (p=0.04), dementia (p=0.03).

^G^- ANOVA, F=0.78, p=0.62, post hoc analysis of LSD revealed a tendency of a statistical difference between PD and MCI (p=0.06), dementia (p=0.07).

^H^- Pearson χ2=50.9, p<0.001.

^I^- Pearson χ2=17.7, p=0.023.

^J^- Pearson χ2=16.5, p=0.04.

Abbreviations: SN- substantia nigra, R-right side, L-left side, LV- lateral ventricle, PD- Parkinson‘s disease, ET- essential tremor, APS- atypical parkinsonian syndromes, HDP- hereditary degenerative parkinsonism, SP- secondary parkinsonism, MCI- mild cognitive impairment, +-hyperechogenic, N.-nucleus, (-i), S.-substantia.
